# Potential mechanisms of acupuncture for neuropathic pain based on somatosensory system

**DOI:** 10.3389/fnins.2022.940343

**Published:** 2022-09-20

**Authors:** Xin Ma, Wen Chen, Na-Na Yang, Lu Wang, Xiao-Wan Hao, Chun-Xia Tan, Hong-Ping Li, Cun-Zhi Liu

**Affiliations:** ^1^School of Acupuncture-Moxibustion and Tuina, Shandong University of Traditional Chinese Medicine, Jinan, China; ^2^School of Acupuncture-Moxibustion and Tuina, International Acupuncture and Moxibustion Innovation Institute, Beijing University of Chinese Medicine, Beijing, China

**Keywords:** neuropathic pain, acupuncture, ion channels, glial cells, descending pain control system, somatosensory system

## Abstract

Neuropathic pain, caused by a lesion or disease of the somatosensory system, is common and distressing. In view of the high human and economic burden, more effective treatment strategies were urgently needed. Acupuncture has been increasingly used as an adjuvant or complementary therapy for neuropathic pain. Although the therapeutic effects of acupuncture have been demonstrated in various high-quality randomized controlled trials, there is significant heterogeneity in the underlying mechanisms. This review aimed to summarize the potential mechanisms of acupuncture on neuropathic pain based on the somatosensory system, and guided for future both foundational and clinical studies. Here, we argued that acupuncture may have the potential to inhibit neuronal activity caused by neuropathic pain, through reducing the activation of pain-related ion channels and suppressing glial cells (including microglia and astrocytes) to release inflammatory cytokines, chemokines, amongst others. Meanwhile, acupuncture as a non-pharmacologic treatment, may have potential to activate descending pain control system *via* increasing the level of spinal or brain 5-hydroxytryptamine (5-HT), norepinephrine (NE), and opioid peptides. And the types of endogenously opioid peptides was influenced by electroacupuncture-frequency. The cumulative evidence demonstrated that acupuncture provided an alternative or adjunctive therapy for neuropathic pain.

## Introduction

Neuropathic pain, caused by a lesion or disease of the somatosensory system, is one of the most intractable human complaints (Jensen et al., 2011). Patients commonly experienced spontaneous pain and/or evoked pain. The former is described as shooting, lancinating or burning pain and the latter is characterized by hyperalgesia to mechanical and cold stimulus (Bouhassira, 2019; Bannister et al., 2020). In addition to the obvious pain-related suffering, neuropathic pain may lead to negative effects such as depression, anxiety, and reduce the quality of life in patients (Laumet et al., 2015). Epidemiological studies have shown that more than 7% of the general population who undergo neuropathic pain, accounting for 20 to 25% of individuals with chronic pain (Torrance et al., 2006; Bouhassira et al., 2008). The high morbidity brought enormous psychological and economic burden to patients, families, and society. However, treatment of neuropathic pain has been extremely challenging, and treatment options are often unmanageable and limited due to the side effects and tolerability (Vranken, 2012; Finnerup et al., 2015). Conventional options to manage neuropathic pain leave much to be desired and more complementary therapies are sorely needed.

The somatosensory system consists of a number of neural pathways that carry various senses from the starting point in skin, muscles, tendons, and internal organs to the central nervous system and ultimately to consciousness (Gomez-Ramirez et al., 2016). Neuropathic pain is a direct consequence of alterations in the somatosensory system (Laedermann et al., 2013). Acupuncture as a non-invasive strategy for nerve stimulation, was a valuable therapy to improve neuropathic pain with a low incidence of adverse events (Barnes et al., 2008; Kelly and Willis, 2019). The somatosensory system mediates the sensation of *de qi* in acupuncture and plays an important role in the analgesic mechanism of acupuncture (Su et al., 2014). Aβ, Aδ, and C fibers are the most important types of primary afferent nerves in transmitting the acupuncture signal (Huo R. et al., 2020). The pain relief effects of acupuncture are also mediated by activity in the brain and spinal cord (Han, 2004). Along with the increasing application of acupuncture, the analgesic mechanisms of acupuncture through the somatosensory system has been increasingly discovered and progressively confirmed. This review synthesized relevant studies to gain a comprehensive understanding of the production, transmission and processing of acupuncture-like signals from the periphery to the central nervous system.

## Materials and methods

### Search strategy

We searched PubMed, Web of Science, and Embase for available information describing issues related to acupuncture and neuropathic pain. The searches identified English language papers published from 2,000 up to the present time. Keywords included [“Acupuncture” or “Electroacupuncture” or “EA”] and [“Neuropathic pain” or “Peripheral neuropathic pain” or “Chronic neuropathic pain after peripheral nerve injury” or “Trigeminal neuralgia” or “Painful radiculopathy” or “Postherpetic neuralgia” or “painful diabetic neuropathy” or “Neuropathy after radiotherapy” or “Neuropathy after chemotherapy” or “Central post-stroke pain” or “Spinal cord injury pain” or “Multiple sclerosis pain” or “Parkinson’s disease pain”].

### Literature selection

The inclusion criteria were designed as follows: (1) Research focused on acupuncture for neuropathic pain. (2) The intervention measures of the treatment group were acupuncture (manual acupuncture or electroacupuncture). (3) Literatures contained clinical research, systematic review/meta-analysis, and basic research. Meanwhile, studies that did not meet the above mentioned criteria were excluded.

**FIGURE 1:**
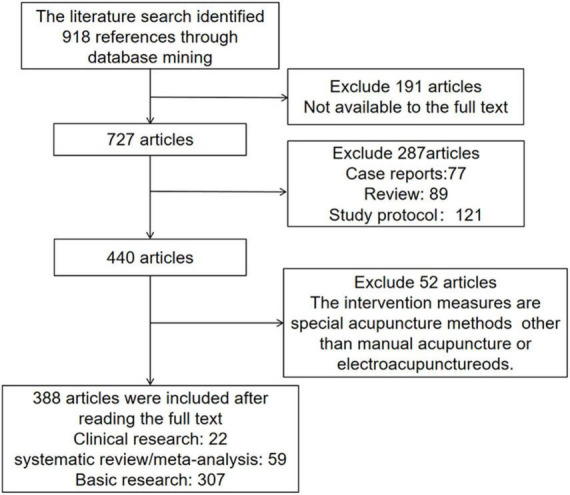
Flow chart of search and filter process.

Searches retrieved 918 articles. After careful evaluation of the data, we sought to summarize the information identified through the literature search. The search was performed by screening the reference lists of articles that met our inclusion criteria based on the titles and abstracts. Of these articles, we excluded 191 articles due to the absence of the full text, leaving 727 articles. Then, we excluded 287 articles including 77 case reports, 121 study protocols, and 89 review articles. Through the title and abstract, we excluded 52 articles that intervention measures were special acupuncture methods other than manual acupuncture or electroacupuncture. Finally, 388 articles were included after reading the full text, including 22 clinical studies, 59 meta-analyses/systematic reviews, and 307 basic studies. A flow chart of the search and filter process is shown in Figure 1.

## Application of acupuncture for neuropathic pain

### Acupuncture manipulations

Manual acupuncture (MA) and electroacupuncture (EA) were the two most common interventions for pain with acupuncture. In MA, the acupuncture needles are inserted into the acupoints and twisted up and down by hand. MA emphasizes the occurrence of *de qi* sensations, which can be induced by correct and effective manual manipulation (Spaeth et al., 2013). In EA, stimulating current is delivered to acupoints *via* the needle connected to an electrical stimulator. The therapeutic benefit of EA depends on the frequency, current amplitude, and pulse width of stimulation. Changing manipulations of MA or parameters of EA may produce different therapeutic effects (Xu et al., 2020). Comparing the changes in pain thresholds for different frequencies of rotational MA, strong stimulation of MA (4 r/s MA) was more effective than mild MA (2 r/s MA) on pain model rats, which was associated with C fiber activation (Song et al., 2021). The analgesia induced by 2 Hz EA was mediated by the endomorphin and that of 100 Hz EA by dynorphin (Han, 2004). In the neuropathic pain model rats, low-frequency (2 Hz) EA had a considerably greater effect on mechanical and thermal pain than high-frequency (100 Hz) EA (Sun et al., 2002). 2/100 Hz (at 2 Hz and 100 Hz frequencies alternately) stimulation increased the release of both endomorphin and dynorphin. It was thus obvious that a proper combination of different frequencies might produce a maximal release of a cocktail of neuropeptides for better therapeutic effects (Han, 2003). Despite of evidence supported the effectiveness of acupuncture for neuropathic pain and showed benefits of MA and EA. It was controversial whether EA does more effective than MA. EA results in more reproducible stimulation and may be substantially more advantageous than MA in continuous stimulation and reducing response times (Zhao et al., 2019). However, another study suggested that EA was not superior to MA treatment. Both therapies had similar efficacy in reducing chronic pain (Comachio et al., 2020).

### Acupoints for neuropathic pain

**FIGURE 2:**
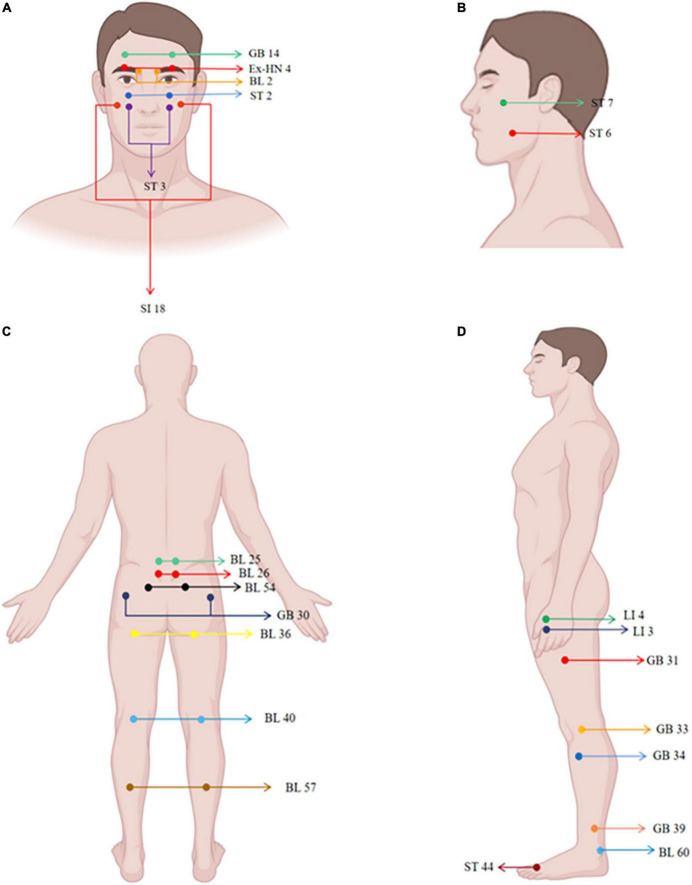
Human maps of acupoints used in neuropathic pain studies. The locations of acupoints are marked in the figure.

There are several ways to select acupoints for neuropathic pain: local, regional, and distal points (Millstine et al., 2017). Common local points are where patients feel the most intense pain or pressure (or both) along the pain distribution, in light of the fact that the location of these acupoints are anatomically identical to those of humans. E.g., trigeminal neuralgia take Yuyao (Ex-HN 4), Cuanzhu (BL 2), and Yangbai (GB 14) (Figure 2A), which are located around the eyes (Gao et al., 2019); radiculalgia (lumbar) take Dachangshu (BL 25), and Guanyuanshu (BL 26) (Figure 2C) which parallel to the fourth and fifth lumbar spinous processes (Yu et al., 2021). Regional points are taken by following the meridians or according to the nerve distribution characteristics of the pain area. For trigeminal neuralgia, maxillary branch includes Quanliao (SI 18), Sibai (ST 2), and Juliao (ST 3) (Figure 2A), and mandibular branch includes Xiaguan (ST 7) and Jiache (ST 6) (Figure 2B), which all belonged to the stomach meridian of foot-yangming (Yu et al., 2021). Depending on the distribution of pain, such as persistent lower limb pain caused by nerve root compression, pain confined to the side of the affected leg will be treated through acupoints on the gallbladder meridian, including Huantiao (GB 30), Fengshi (GB 31), Xiyangguan (GB 33), Yanglingquan (GB 34), and Xuanzhong (GB 39) (Figures 2C,D). Pain confined to the posterior part of the affected leg will be treated through points on the bladder meridian, including Zhibian (BL 54), Chengfu (BL 36), Weizhong (BL 40), Chengshan (BL 57), and Kunlun (BL 60) (Figures 2C,D; Yu et al., 2021). Distal regions, such as Neiting (ST 44), Hegu 4 (LI 4), and Sanjian (LI 3) (Figure 2D), are taken for trigeminal neuralgia (Gao et al., 2019). It is an acupoint selection method based on traditional Chinese medicine theory. The location of acupoint is determined using basic theoretical frameworks (e.g., traditional Chinese medicine, TCM) or anatomical structures (e.g., innervation). Currently, acupuncturists tend to use a hybrid approach when providing TCM-based acupoint localization, and they may combine their practice with localized treatments based on current anatomical knowledge, such as the principles of acupoint selection mentioned earlier.

### Clinical evidence for acupuncture on neuropathic pain

**FIGURE 3:**
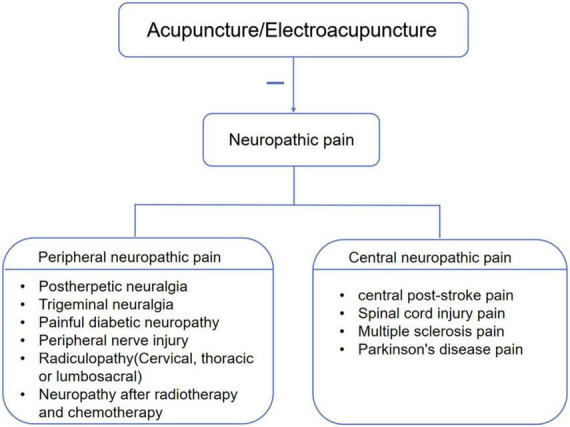
Acupuncture inhibits neuropathic pain induced by various diseases.

A variety of diseases including peripheral and/or central nerve injury, neuropathic pain induced by diabetes, anticancer chemotherapy, post-stroke pain and Parkinson’s have been used to study the effect of acupuncture on neuropathic pain (Figure 3; Becker et al., 2017; Fan et al., 2018; Zhi et al., 2018; Lovaglio et al., 2019; Yu et al., 2019; Wang et al., 2020; Edwards and Shaw, 2021; Zhang et al., 2021; Sollie et al., 2022). Table 1 listed acupuncture treatments for neuropathic pain caused by different diseases. In addition to the difference in the treatment of acupoints, the depth of acupuncture, the needle manipulation and the course of treatment may also be varied. These methods were not the only ones that physicians also made determinations based on their clinical experience. With the deepening of clinical research, the efficacy of acupuncture on neuropathic pain has been confirmed by several systemic reviews and meta-analyses, which were listed in Table 2 (Heo et al., 2013; Dimitrova et al., 2017; Hu et al., 2019; Liu et al., 2019; Pei et al., 2019; Yun et al., 2020; Cui et al., 2021; Yu et al., 2021). A study described the effectiveness and safety of acupuncture for the treatment of chemotherapy-induced peripheral neuropathy. After 8 weeks of treatment and follow-up, the acupuncture group showed a greater reduction in pain score than the vitamin B1 or gabapentin group. Moreover, the nerve conduction study was improved best in the acupuncture group and no adverse events were observed (Iravani et al., 2020). Based on currently available evidences, acupuncture appears more effective than pharmacotherapy or surgery with high degree of safety for improving neuropathic pain (Huang Z. et al., 2019; Yu et al., 2019; Iravani et al., 2020; Lee et al., 2020).

**TABLE 1:** Acupuncture methods for neuropathic pain caused by different diseases.

Condition	Acupoints	Acupuncture methods	Duration of treatment	References
Trigeminal neuralgia	Neiting (ST 44), Hegu 4 (LI 4), Sanjian (LI 3), ophthalmic branch (Yuyao, Ex-HN 4; Cuanzhu, B 2; Yangbai, GB 14), maxillary branch (Quanliao, Sl 18; Sibai, ST 2; Juliao, ST 3), and mandibular branch (Xiaguan, ST 7; Jiache, ST 6; Extraordinary point, Ex)	The penetration depth was 25 to 50 mm in the muscle.	The retention time for the needles was 20 min and one session per week for a total of 10 weekly sessions.	Gao et al., 2019
Sciatica	The bilateral acupoints of Dachangshu (BL 25), Shenshu (BL 23), Weizhong (BL 40), and Chengshan (BL 57)	The needles were inserted 30–70 mm into the acupoints slowly and vertically. Twirling, lifting, and thrusting manipulates were performed tenderly and evenly three times in order to reach the *de qi* sensation.	12 sessions of treatment (30 min each) for 4 weeks (three times a week).	Huang Z. et al., 2019
Painful diabetic neuropathy	Zusanli (ST 36), Feishu (BL 13), Pishu (BL 20), Sanyinjiao (SP 6), and Yinlingquan (SP 9), Xuanzhong (GB 39), Taichong (LR 3) and Zulinqi (GB 41)	Needles will be inserted perpendicularly and stimulated only in the beginning to achieve a *de qi* sensation and then will be left in place.	12 sessions administered over a period of 8 weeks (preferably 2 sessions in each of the first 4 weeks, followed by 1 session per week in the remaining 4 weeks).	Lee S. et al., 2013
Postherpetic neuralgia	Jiaji (Ex-B2) and Ashi points	A filiform needle, 0.25–0.30 mm in diameter, 25–40 mm in length, is stimulated with an electrical current	20–30 min in each session	Ruengwongroj et al., 2020
Spinal cord injury pain	Changqiang (GV 14), Jiaji (Ex-B2), et al.	Needles were inserted to a depth of 15 to 30 mm and left in place for 20 min	15 sessions of acupuncture over a 7^1/2^-week period	Nayak et al., 2001
Chemotherapy-induced peripheral neuropathy	Qihai (CV 6), Baihui (GV 20), Bilateral Zusanli (ST 36), Sanyinjiao (SP 6), Hegu (LI 4), Quchi (LI 11), and Taichong (LR 3) as the general points and bilateral Bafeng (EX-LE 10) and Baxie (EX-UE 9)	Needles (0.25×0.40 mm) were inserted perpendicularly at the depth of 5–15 mm acupoints, with proper needling manipulation to induce “de qi” (the arrival of qi).	3 times per week for 4 weeks (20 min in each session)	Iravani et al., 2020

**TABLE 2:** Efficacy of acupuncture for neuropathic pain.

Authors	Journal	Type of study	Sample size	Disease types	Conclusion
Cui et al. (2021)	Complement Med Res	Systematic review and meta-analysis	915	Trigeminal neuralgia	Acupuncture may be effective for patients with trigeminal neuralgia.
Yu et al. (2021)	J Clin Pharm Ther	Systematic reviews	8378	Diabetic peripheral neuropathy	Acupuncture appears to have an effect on painful diabetic neuropathy, effectively improving nerve conduction and clinical symptoms.
Pei et al. (2019)	J Pain Res	Meta-analysis	498	Postherpetic neuralgia	Acupuncture may reduce pain intensity, relieve anxiety and improve quality of life in patients with postherpetic neuralgia.
Liu et al. (2019)	Front Neurol	Systematic review and meta-analysis	3,184	Central post stroke pain	Adding acupuncture to conventional rehabilitation treatment for post-stroke pain is superior to rehabilitation treatment alone.
Dimitrova et al. (2017)	J Altern Complement Med	Meta-analysis	680	Peripheral neuropathy	Acupuncture is beneficial in some peripheral neuropathies.
Ma et al. (2015)	J Neurotrauma	Systematic review and meta-analysis	35	Spinal cord injury pain	Acupuncture may be an effective treatment for reducing chronic pain in patients with spinal cord injury.

A majority of acupuncturists emphasized *de qi* which is a feeling of numbness, fullness, and sometimes soreness, when they performed acupuncture treatments. It seemed that acupuncture analgesia was manifest when the *de qi* feeling occured in patients following manipulation of acupuncture (Hui et al., 2005). The complete somatosensory system was the prerequisite for the appearance of *de qi* (Cao, 2002). Previous research confirmed that the *de qi* sensation and pressure pain threshold increased according to the depth and rotation of acupuncture (Choi et al., 2013). Treatment for chronic neuropathy pain usually lasts more than 4 weeks. Acupuncture may be a slow-acting agent and has a specific pattern of the dynamics for the entire coupled nervous system. The therapeutic effect of acupuncture is a gradual accumulation process, that is, as the number of acupuncture courses increases, the therapeutic effect gradually increases (Bai et al., 2009). Therefore, the above factors should be considered in acupuncture analgesia.

## Animal models of neuropathic pain

Animal models of neuropathic pain are critical for understanding the underlying mechanism and further development of acupuncture therapy. A variety of neuropathic pain animal models which induced by central or peripheral nerve injury have been used to study the acupuncture mechanism (Table 3; Zhang Y. et al., 2018; Li et al., 2019b; Huo B.B. et al., 2020; Du et al., 2021; Jang et al., 2021; Jiang et al., 2021; Wei et al., 2021; Xu et al., 2021; Zou et al., 2021). In addition to animal models of nerve ligation, there are injectable chemotherapy drug-induced neuropathy, post-herpetic neuralgia, and diabetes-induced peripheral nerve injury (Colleoni and Sacerdote, 2010). The former is caused by a primary injury or dysfunction of the nervous system, while the latter is caused by diseases such as diabetes, shingles, and cancer chemotherapy. Although every model possesses its own unique characteristics, different etiologies of neuropathic pain appear to lead to similar behavioral endpoints.

**TABLE 3:** Animal models of neuropathic pain.

Model	Mode of induction	Disease types	Peripheral/Central
partial sciatic nerve ligation (PSNL)	By ligation of 1/3 or 1/2 of sciatic nerve	Peripheral nerve injury	Peripheral
chronic constriction injury (CCI)	By ligation of sciatic nerve	Peripheral nerve injury	Peripheral
spinal nerve ligation (SNL)	By ligation of L5/L6 of spinal cord	Peripheral nerve injury	Peripheral
Spared nerve injury (SNI)	By ligation of peroneal and tibial nerves	Peripheral nerve injury	Peripheral
Brachial plexus avulsion injury (BPAI)	By damaging the dorsal and ventral nerve rootlets of C5-T1	Peripheral nerve and spinal cord injury	Peripheral and Central
Spinal cord injury (SCI)	By transecting T10 spinal cord	Spinal cord injury pain	Central
Chemotherapy-induced peripheral neuropathy (CIPN)	By i.p. injecting anti-cancer agents (vincristine, cisplatin, oxaliplatin, paclitaxel)	Neuropathy after chemotherapy	Peripheral
Postherpetic neuralgia (PHN) model	By injecting 10 μL PLVX-IRES-Zsgreen1-Mir-223-3p into the lumbar spine between L5 and L6	Postherpetic neuralgia	Peripheral
chronic constriction injury to infra-orbital nerve	By ligation of infra-orbital nerve	Trigeminal Neuralgia	Peripheral
Diabetes-induced neuropathic pain	By persistenting hyperglycemia-induced changes in the nerves	Painful diabetic neuropathy	Peripheral

## Acupuncture mechanisms on neuropathic pain

**FIGURE 4:**
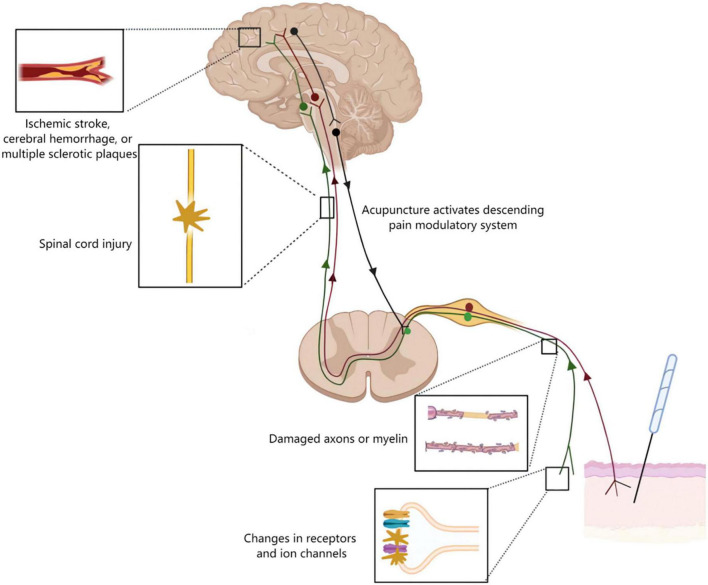
Acupuncture mechanisms on neuropathic pain. Neurological damage from peripheral to the cortical brain may lead to neuropathic pain. The figure shows four typical examples of lesions. Alterations in receptors and ion channels impact neuronal function, resulting in spontaneous (ectopic) activity and pain. Along the peripheral nerve different types of lesions may damage either the entire nerve or selectively the axons or myelin causing axonal or demyelinating neuropathies, respectively. Lesions of the spinal cord or brain as seen for example following traumatic injury, ischemic stroke, cerebral hemorrhage, or multiple sclerotic plaques may lead to central neuropathic pain. Acupuncture inhibited pain transmission *via* the somatosensory system, and activated the descending pain control system.

Neuropathic pain is divided into two major categories: peripheral and central, depending on the location of the lesion or disease (Finnerup et al., 2016; Scholz et al., 2019; Figure 4). At the peripheral level, alterations in receptors and ion channels impact neuronal function, resulting in spontaneous (ectopic) activity and pain (Khan et al., 2019). The pathological hallmarks of different types of peripheral nerve lesions have individual characteristics. Some may damage to the entire nerve, causing axonal neuropathy; others may damage to part of the axon or myelin sheath, causing demyelinating neuropathy (Hoffmann et al., 2008; Held et al., 2019). In the central nervous system, there are a variety of conditions that can cause central neuralgia, including damage to the spinal cord or brain, such as trauma, ischemic stroke, cerebral hemorrhage, or multiple sclerotic plaques (Apkarian et al., 2005; Siddall and Middleton, 2015). The mechanism of acupuncture for neuropathic pain is mediated by the somatosensory system (Figure 4). Acupuncture modulated the alterations of receptors and ion channels, inhibited activation of protein kinases and glia and activated the descending pain control system (Goldman et al., 2010; Li et al., 2013; Xu et al., 2020). This review synthesized these studies to provide a comprehensive understanding of how acupuncture alleviates pain through the somatosensory system.

### Acupuncture modulates the receptors and ion channels in the periphery

Peripheral nerve endings perceive nociceptive stimuli and activate pain pathways. In order for this interaction to happen, mechanical or other stimuli may affect the cytoplasmic membrane potential of axon which as soon exceeds a certain threshold level triggering action potentials. Nociceptors sense thermal, mechanical, and chemical stimuli through the expression of different ion channels such as the transient receptor potential (TRP) family of ion channels as well as ATP-gated purinergic channels (P2X). At this point, diverse types of voltage-gated sodium channels come into play to amplify transient receptor potentials and thus reach depolarization levels sufficient to initiate action potentials (Bannister et al., 2020; Sharif et al., 2020). Transient receptor potential vanilloid 1 (TRPV1) belongs to the family of TRP, that are intensively expressed in the peripheral nervous system and involved in a variety of physiological and pathophysiological processes in mammals (Nilius et al., 2007; Mickle et al., 2015). There is pharmacological evidence that blocking TRPV1 channel, alleviates neuropathic hypersensitivity in rodent models (Basso and Altier, 2017). P2X, specifically the C-fiber localized P2X3 receptor (P2X3R) subtypes, are expressed in the dorsal root ganglion (DRG) and involved in the initiation and maintenance of neuropathic pain (Tang Y. et al., 2016; Khan et al., 2019). Besides, P2X4 and P2X7 in DRG were also involved in thermal nociceptive hypersensitivity (Masoodifar et al., 2021). Voltage-gated sodium channels Na_*v*_1.1, Na_*v*_1.6, Na_*v*_1.7, Na_*v*_1.8, and Na_*v*_1.9 are expressed in peripheral sensory neurons in different patterns and function as key regulators of sensory nerve excitability (Bennett et al., 2019). Mutations in voltage-gated sodium channels are associated with a variety of pain disorders. In neuropathic conditions, Na_*v*_1.8 is most highly expressed in small-diameter neuron subtypes (Dib-Hajj and Waxman, 2019). Another family of excitatory channels associated with neuropathic pain is hyperpolarization-activated and cyclic nucleotide-gated (HCN) channels. HCN2 was specifically deleted in nociceptors expressing Na_*v*_1.8 in mice, but nerve lesion did not cause hyperalgesia to thermal or mechanical stimuli (Emery et al., 2011). HCN2 antagonist attenuated neuropathic hypersensitivity in neuropathic rats and inhibited spontaneous activity of C-nociceptors, but not Aβ fiber (Djouhri et al., 2018).

**FIGURE 5:**
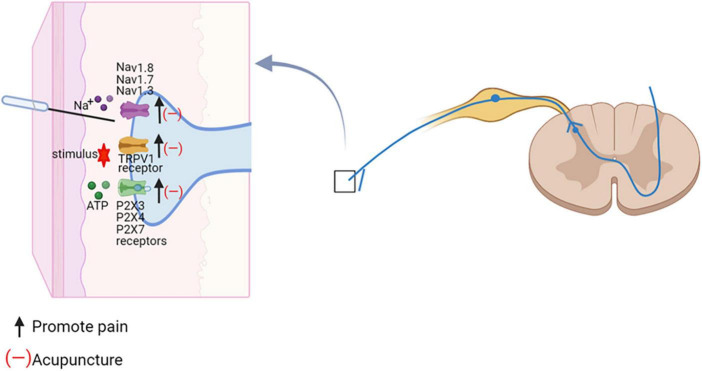
Acupuncture regulates ion-channel dysregulation in neuropathic pain. The effect of acupuncture on the spinal cord. Na+, sodium ion; Navs, Voltage-gated sodium channels; TRPV1, TRP vanolloid 1; ATP, adenosine triphosphate; P2X3, ATP-gated purinergic channels 3.

Electroacupuncture (EA) effectively reduced nociceptive sensitization in spared nerve injury (SNI) and spinal nerve ligation (SNL) by downregulating the expression ratio of TRPV1 in DRG (Fang et al., 2021). Administration of TRPV1 agonists reversed EA analgesia (Jiang et al., 2013; Du et al., 2021). Ca^2+^ imaging revealed that TRPV1 channel activity was increased in DRG neurons of paclitaxel-treated rats, whereas EA suppressed the increased TRPV1 channel activity. Pharmacological blockade of TRPV1 was similar to the analgesic effect of EA on pain allergy, while capsaicin reversed the effect of EA (Li et al., 2019b). EA might inhibit the activation of P2X3Rs in neuropathic pain and block primary afferent transmission mediated through P2X3Rs to alleviate mechanical and thermal nociceptive sensitization (Fei et al., 2020). Additionally, EA was more potent in reducing both mechanical allodynia and thermal hyperalgesia in combination with intrathecal A-317491 (a selective P2X3 and P2X2/3 receptor antagonist) (Wang et al., 2014). Therefore, EA and A-317491 might potentially have an additive effect in inhibiting the transmission of pain mediated by the P2X3 receptor. The protein levels of P2X4 and P2X7 in diabetes-induced neuropathy rats were significantly increased. 2 Hz EA improved the paw withdrawal latency and reduced the expression of P2X4 and P2X7 in DRG (Hu et al., 2022). EA attenuated Na_*v*_1.7 and Na_*v*_1.8 protein expression levels in the DRG during painful states (Yen et al., 2018). Na_*v*_1.3 was lacking in DRG neurons of normal adult rats, but was highly expressed in damaged sensory neurons (Waxman et al., 1994). EA diminished spinal cord injury (SCI)-induced upregulation of Na_*v*_1.3 (Liu and Wu, 2017). EA also reduced mechanical allodynia and face-grooming in trigeminal neuropathic pain rats through downregulation of HCN expression in the gasserian ganglion (Yang et al., 2019). These results suggested that acupuncture blocks pain-related ion channels and increases pain thresholds (Figure 5).

The available evidence indicated that afferent nerve fibers and different receptors in the acupoints might played a key role in mediating the effects of acupuncture. Acupuncture inhibited neuronal activity caused by neuropathic pain, through reducing pain-related ion channels and receptors activation. At present, however, the specific pathway of changes in receptors and ion channels mediated by acupuncture stimulation points cannot be explained. More types of sham controls need to be employed to thoroughly evaluate the effects of acupoint specificity in future studies.

### Acupuncture inhibits activation of protein kinases and glia in the spinal cord

The spinal cord neuronal activity caused by neuropathic pain is partially attributed to increased synaptic efficacy. Ionic and metabotropic glutamate receptors exhibit phosphorylation or translocation changes, resulting in increased excitatory postsynaptic potential (EPSP) frequency and amplitude (Kiyoyuki et al., 2015; Hildebrand et al., 2016). Multiple protein kinases have been implicated in regulating neuronal plasticity and pain sensitization following intense noxious stimuli or injuries. There is increasing evidence suggesting that serine/threonine kinases family especially Protein kinase A (PKA), Protein kinase C (PKC), mitogen-activated protein kinases (MAPKs) (Ma et al., 2021), is critical for the induction and maintenance of pain hypersensitivity after injuries (Ji et al., 2007). Extracellular signal regulated kinase (ERK) (Kondo and Shibuta, 2020), p38 mitogen-activated protein kinases (p38 MAPK) (Lin et al., 2014), and calmodulin-dependent protein kinase II (CaMKII) (Qian et al., 2019) are downstream to many kinases. These kinases are activated in primary sensory and dorsal horn neurons by nociceptive activity, contributing to the induction and maintenance of pain sensitization (Choi et al., 2019). Compared with the normal rat, more PKA-positive cells were observed in the spinal dorsal horn of SNL rat (Wu et al., 2021). PKC activation depolarized unmyelinated afferent neurons, which enhance currents in afferent neurons activated by nociceptive stimuli, and PKC inhibitors blocked sensitization of afferent neurons (Velázquez et al., 2007). p38 MAPK and ERK are present in spinal dorsal horn, and their inhibitors inhibit neuropathic pain (Inoue and Tsuda, 2018). The activation pattern of ERK in the spinal cord correlated with neuropathic pain behavior at different time points after SNL. Intrathecal injection of the non-competitive ERK inhibitor PD98059 attenuated SNL-induced mechanical nociceptive hypersensitivity (Kondo and Shibuta, 2020). Inhibition of spinal CaMKII expression has been shown to prevent thermal hyperalgesia and mechanical allodynia (Fang et al., 2002).

Electroacupuncture (EA) reduced the expression of p38 MAPK and inhibited pain transmission in rat spinal cord dorsal horn (Wei and Hsieh, 2020; Jin et al., 2021). PKA expression levels are elevated and involved in neuropathic pain by activating the p38 MAPK pathway to mediate apoptosis in spinal cord cells (Deng et al., 2020). EA exerted analgesic effects by decreasing the expression of PKA in SNL model rats (Wu et al., 2021). 2 Hz EA reduced the expression of P2X3 receptors by inhibiting the PKC pathway thus relieved pain (Zhou et al., 2018), CaMKII is crucially involved in synaptic plasticity and long-term potentiation (LTP) (Luo et al., 2014). It was found that EA reduced p-CaMKII levels in the spinal cord and was blocked by pretreatment with 5-hydroxytryptamine (5-HT) 1A receptor antagonists, suggesting that 5-HT1A receptors were involved in the inhibitory effect of EA on spinal p-CaMKII (Zhang Y. et al., 2018). These studies clearly showed that acupuncture blocked multiple protein kinases activation to reduce spinal cord neuronal activity in painful conditions and achieved pain relief.

Proliferation, shape change and activation of microglial populations in the spinal dorsal horn has been reported in several models of neuropathic pain (Ji et al., 2016). Diverse ensuing changes in the transcriptional and secretory profile of microglia have been linked to neuropathic pain, including release of inflammatory factor, ATP, chemokines, amongst others (Inoue and Tsuda, 2018). Moreover, astrocyte activation further promotes neuronal activity (Ji et al., 2016). It is known that p38 MAPK and ERK play important roles in the maintenance of neuropathic pain (Deng et al., 2020; Kondo and Shibuta, 2020). P-p38 MAPK and p-ERK upregulation are localized to activate microglia within the dorsal horn of the lumbar region after spinal cord injury. CX3C chemokine fractalkine (CX3CL1) released from damaged neurons activates CX3C-chemokine receptor 1 (CX3CR1) on microglial cells and leads to tumor necrosis factor α (TNF-α), and Interleukin-1β (IL-1β) secretion *via* p38 MAPKs/ERK. Released IL-1β and TNF-α acts on spinal dorsal horn neurons to enhance glutamate excitatory synaptic transmission and decreaseγ-aminobutyric acid (GABA)-mediated and glycine-mediated synaptic inhibition (Gim et al., 2011; Inoue and Tsuda, 2018). Furthermore, ERK in activated microglia mediates the release of prostaglandin E2 (PGE2), which binds to prostaglandin E receptor 2 (EP2) expressed in spinal cord neurons, inducing a change in their excitatory state and thus causing neuropathic pain (Zhao et al., 2007). Spinal brain-derived neurotrophic factor (BDNF) is a key neuromodulator of pain transmission, and P2X4R activates spinal microglia to induce p38 MAPK phosphorylation to release BDNF, which transmits noxious signals to layer I neurons, thereby contributing to the pathogenesis of pain (Cappoli et al., 2020).

**FIGURE 6:**
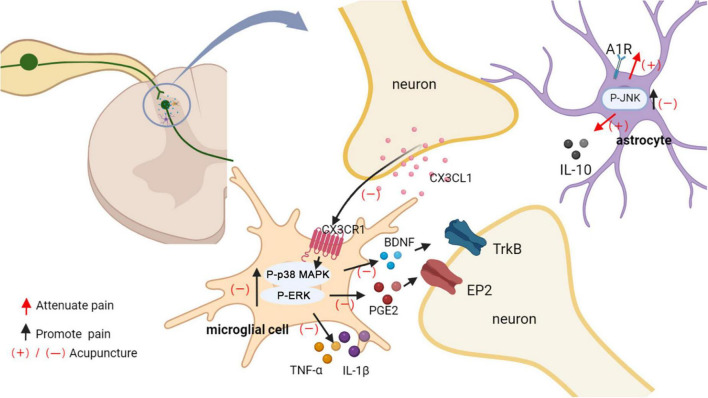
Acupuncture affects glial activation. EA down-regulated the neuronal chemokine CX3CL1 and prevented the p38 MAPK/ERK signaling pathway, leading to reduced release of TNF-α and IL-1β, BDNF, PGE2. Released IL-1β and TNF-α acts on spinal dorsal horn neurons to enhance the neuronal excitability and inflammatory response. PGE2 and BDNF bind to spinal cord neuronal EP2 and Trk B, respectively, inducing a change in their excitatory state and thus causing neuropathic pain. EA induced an increase in adenosine levels in the spinal cord, which in turn activated astrocyte A1Rs to produce an analgesic effect. Additionally, analgesic effect of acupuncture was mediated in part through inhibiting the activation of JNK and promoting the release of IL10 in astrocytes.

Microglia and astrocytes, are important targets for acupuncture analgesic (Figure 6). EA reduced mechanical and thermal pain in rat models of neuropathic pain by preventing microglia, astrocyte activation (Liang et al., 2016). EA and intrathecal injection of the glial metabolism suppressant fluorocitrate might synergize against pain (Sun et al., 2006). Therefore, the inactivation of glial cells may be partly responsible for the acupuncture analgesic. In a SCI model, acupuncture applied to GB34 inhibited p38 MAPK and ERK phosphorylation in microglia of L4-5 spinal cord (Choi D. C. et al., 2012). EA down-regulated the neuronal chemokine CX3CL1, which acted on CX3CR1 in microglia, and prevented the p38 MAPKs/ERK signaling pathway, leading to reduce the release of inflammatory cytokines, resulting in pain relief (Li et al., 2019a). Acupuncture attenuated the ERK-dependent PGE2 releasing from activated microglia (Choi D. C. et al., 2012). EA prevented BDNF binding to spinal cord neuronal tyrosine kinase receptor B (Trk B) by decreasing microglia activation and BDNF expression, thereby reducing nociceptive hyperalgesia and neuropathic pain (Tu et al., 2018).

Adenosine is present at the extracellular space within the spinal cord dorsal horn and engaged in the processing of nociceptive sensory signals. Systemic or spinal administration of exogenous adenosine produces a potent analgesia against pathological pain. In rat spinal cord slices, adenosine increases postsynaptic inhibitory currents mediated by glycine receptors (GlyRs), and this synaptic potentiation is dependent on activation of adenosine A1 receptors (A1Rs) (Bai et al., 2017). Another study found that spinal A1R contributed to the inhibitory effects of EA on astrocyte activation as well as TNF-α upregulation (Zhang M. et al., 2018). The c-Jun N-terminal kinase (JNK), a major member of the MAPK family, has been shown to play a key role in intracellular signaling and contributes to central sensitization of chronic pain. Peripheral inflammation or nerve injury leads to JNK activation in spinal astrocytes. Activation of the JNK pathway lead to the production and release of several pro-inflammatory cytokines that play an important role as biological mediators in chronic pain (Wang et al., 2017).

It has been shown that EA first downregulated microglia activation (after 2 days of EA) and then astrocyte activation (after 1–2 weeks of EA treatment) (Wang et al., 2018). A1Rs expression in the L4–6 spinal segments were increased by EA, indicating that EA induced an increase in adenosine levels in the spinal cord, which in turn activated astrocyte A1Rs to produce an analgesic effect (Dai et al., 2020). In SCI rats, acupuncture inhibited JNK activation in astrocytes at the spinal cord L4-5 level. The level of p-c-Jun, a downstream molecule of JNK, was also decreased by acupuncture. In addition, the number of hypertrophic, activated astrocytes in the L4-5 dorsal horn I-II layers was significantly reduced in the acupuncture-treated group. It was suggested that the analgesic effect of acupuncture was mediated in part through inhibition of JNK activation in astrocytes after SCI (Lee J. Y. et al., 2013). Interleukin-10 (IL-10) is a powerful anti-inflammatory cytokine that improves inflammation and protects damaged nerve. It mainly distributes in the superficial spinal astrocytes. The anti-nociceptive effects of EA were blocked by the spinal IL-10 inhibitor, suggesting EA had a regulatory effect on IL-10 in spinal astrocytes (Dai et al., 2019).

The spinal cord is an important center for mediating the analgesic effects of acupuncture. EA down-regulated the neuronal chemokine CX3CL1 and prevented microglial p38 MAPK/ERK signaling pathway, leading to reduced release of TNF-α and IL-1β, BDNF, PGE2, and reducing the neuronal excitability and inflammatory response. The activation of microglial cells in the ipsilateral spinal cord dorsal horn increased within 1 day of nerve injury; however, astrocytes were activated later than microglial cells and were implicated in the maintenance of mechanical allodynia after spinal nerve injury. In the early and late stages of neuropathic pain, repeated EA therapy inhibited microglia and astrocyte activation, respectively (Liang et al., 2016). These results suggested that inhibition of spinal microglia activation was involved in the early stage of EA analgesia, while inhibition of astrocytes activation was involved in the maintenance of EA analgesia.

### Acupuncture regulates descending pain control system

**FIGURE 7:**
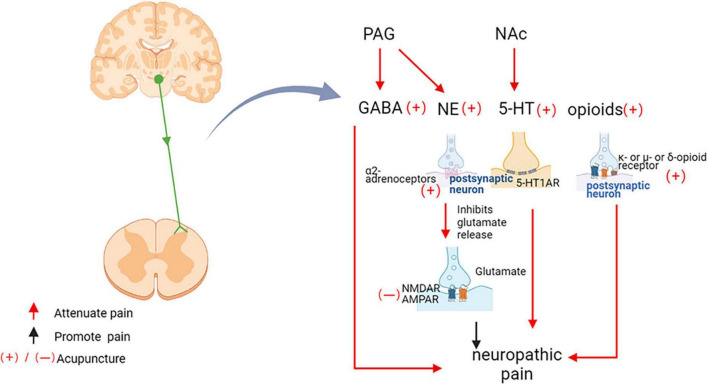
Acupuncture inhibits neuropathic pain through the downstream pathway. PAG, periaqueductal gray matter; nucleus accumbens, NAc; GABA, Gama aminobutyric acid; NE, Norepinephrine; 5-HT, 5-hydroxytryptamine; 5-HT1AR, 5-hydroxytryptamine 1A receptors; 5-HT3R, 5-hydroxytryptamine 3 receptors; NMDAR, *N*-methyl-D-aspartate receptors; AMPA, α-amino-3-hydroxy-5-methyl-4-isoxazolepropionic acid receptors.

It is well-established that the descending pain control system of the midbrain and brainstem regulates the processing of nociceptive information in the spinal cord. The anterior cingulate cortex (ACC) promotes spinal excitatory synaptic transmission leading to nociceptive hyperalgesia. Descending corticospinal tract fibers originating from somatosensory cortex project not only to the spinal ventral horn, but also to the spinal dorsal horn and to the sensory synapse (Liu et al., 2018). Imaging studies in human volunteers showed that the rostral ACC likely mediated analgesia by activation of the periaqueductal gray (PAG) (Eippert et al., 2009). The PAG is known to be an important relay station receiving inputs from higher brain centers for descending modulation of pain through projections to the spinal cord *via* the rostroventral medulla (RVM) and the locus coeruleus (LC) (Alhadeff et al., 2018). In neuropathic pain, acupuncture plays a complex and critical role in the network of descending pathways projecting from brain structures to the spinal dorsal horn (Figure 7). The analgesic effect of descending pain control system relies on endogenous gamma-aminobutyric acid GABA, 5-HT, norepinephrine (NE), and endogenous opioids (Silva et al., 2013).

Gama aminobutyric acid (GABA) is an important inhibitory neurotransmitter. GABAergic projections of interneurons from the brainstem to the spinal cord control spinal cord nociceptive transmission (François et al., 2017). The PAG manages pain through downstream modulation of the spinal dorsal horn, and EA activation of PAG neurons involved the descending pain control system of GABAergic (Fusumada et al., 2007). In the neuropathic pain model induced by chronic constriction injury (CCI), EA up-regulated the level of GABA in the PAG (Huang C. P. et al., 2019). EA at Jiaji (EX-B2) acupoints improved neuropathic pain by increasing protein expression levels of GABA receptors in the spinal cord (Jiang et al., 2018). Using *in vivo* two-photon imaging in mice with chronic systolic injury, it was found that EA therapy systematically modulates Ca^2+^ activity in primary somatosensory cortical neural circuits, including inhibition of excitatory pyramidal neurons, enhancement of GABA-ergic somatostatin positive interneurons, thereby mediating improved mechanical or thermal hypersensitivity (Wei et al., 2021).

5-HT and its receptors are key substances that regulate pain and play a crucial role in EA analgesia. 5-HT released from the nucleus accumbens (NAc) were increased in rats receiving acupuncture, which was observed 20 min after acupuncture treatment and persisted until 40 min after the end of acupuncture (Yoshimoto et al., 2006). Exogenous lateral ventricular 5-HT have analgesic effects that partially mimic the analgesic effects of EA (Chang et al., 2004). Current research indicated that the alleviation of cold nociceptive hypersensitivity by 2 Hz EA was mediated by spinal 5-HT1A and 5-HT3 receptors (Kim et al., 2005). In addition, it has been shown that lateral ventricular injections of 5-HT1A receptors antagonists blocked the analgesic effects induced by low-and high-frequency EA (Chang et al., 2004). Blockade of 5-HT1A receptors in the ventral tegmental area reversed morphine-/dextromethorphan-induced analgesia in pain model rats (Seddighfar et al., 2019). Spinal 5-HT2A receptors (5-HT2AR) mediate the downstream vulnerability of 5-HTergic axons through multiple mechanisms. Inhibition of spinal dorsal horn 5-HT2AR expression prevents mechanical nociceptive hypersensitivity of the face and associated changes in PKCγ^+^ interneuron morphology (Alba-Delgado et al., 2018). However, there are no reports of neuropathic pain relief by acupuncture mediated by 5-HT2A, providing a direction for the next study.

Norepinephrine (NE) and α_2_-adrenergic receptors are widely distributed in the brain and spinal cord, and activation of the NEergic descending pain control system is involved in the anti-nociceptive effects of EA (Silva et al., 2011). Acupuncture at Zusanli (ST 36) and Shangjuxu (ST 37) activated neurons in the PAG to exert anti-nociceptive effects, increased the release of NE from the PAG. The α_1_-, α_2_-, β-adrenoceptors were found to be located in the PAG, and noradrenalin and α_1_-and α_2_-agonists were found to activate the lateral and ventrolateral PAG neurons. These findings suggested that the modulation of pain by EA in PAG involved NE (Murotani et al., 2010). Intrathecal injection of drugs that increase spinal NE utilization promotes the long-term anti-nociceptive effects of EA (Silva et al., 2013). Intrathecal injection of the α_2_–adrenergic antagonist yohimbine reduced EA-induced analgesia in a dose-dependent manner, suggesting that the analgesic effect of EA was dependent on the binding of norepinephrine to α_2_ receptors (Koo et al., 2008).

Endogenous opioids were closely related to the analgesic effect of acupuncture. Acupuncture reduced the SNL-induced hypersensitivity response, which blocked by naloxone, a non-selective opioid receptor antagonist (Cidral-Filho et al., 2011), suggesting that the analgesic effect of acupuncture was dependent on the opioid system. Low-frequency and high-frequency EA activated different types of opioid receptors. Mu-or delta-opioid receptor antagonists blocked the 2 Hz EA anti-mechanical nociceptive hypersensitivity (Kim et al., 2004). 100 Hz EA leaded to the release of dynorphin, which binded to kappa-opioid receptors in the spinal cord and provides pain relief (Huang et al., 2008). However, 2/100 Hz EA activated both mu/delta and kappa opioid receptors, inducing a synergistic analgesic effect that was more effective than constant frequency stimulation (Han, 2003).

Ionic glutamate receptors include *n*-methyl-d-aspartate (NMDA) receptors and α-amino-3-hydroxy-5-methyl-4-isoxazolidinepropanoic acid (AMPA) receptors. Glutamate is the main excitatory neurotransmitter acting on ionotropic glutamate receptors to direct central sensitization (Groc and Choquet, 2006). EA inhibited phosphorylation of AMPA receptor (AMPAR) GluR2 subunit (Tang Y. et al., 2016). Low-frequency EA exerted analgesic effects by modulating the phosphorylation status of spinal NMDA receptor subunits NR1 and NR2B (Jung et al., 2010). EA improved SNI-induced pain behavior and decreased glutamate release in the spinal dorsal horn (Vidal-Torres et al., 2012). 5-HT1BR (Choi I. S. et al., 2012) and α_2_-adrenoceptors (Li and Eisenach, 2001) activation reduced glutamate release from medullary dorsal horn neurons. However, in SNL-induced neuropathic pain rat model, 2 Hz EA activated the endogenous opioid system and induces NMDA receptor-dependent long-term depression (LTD) in the spinal dorsal horn (Xing et al., 2007). The different results may be explained by different acupoints and different parameters of EA.

After the initial injury healing, chronic pain will continue, which is related to brain remodeling (Kuner and Flor, 2016) and the treatment rely on post-therapeutic effect of EA. Brain processing of acupuncture stimuli in neuropathic pain patients or animals may underlie its beneficial effects. Previous study showed that neuropathic pain following brachial plexus avulsion injury (BPAI) induced metabolic connectivity changes significantly among sensorimotor-related areas and pain-related area in bilateral hemispheres (Huo R. et al., 2020). The decreased metabolic connectivity between ipsilateral dorsolateral thalamus and somatosensory cortex was related with BPAI-induced neuropathic pain. EA increased the thermal withdrawal latency of BPAI rat and improved the strength of connectivity among the above regions (Hou et al., 2020). Evidence suggested that neuropathic pain patients responded to acupuncture with more pronounced fMRI signal decrease in the amygdala and signal increase in the lateral hypothalamic area differently than healthy people. Acupuncture coordinated limbic response that included the hypothalamus and amygdala (Napadow et al., 2007). The hypothalamus is an important component of the central descending pain modulatory circuit from the cerebral cortex to the spinal cord (Ossipov et al., 2010), activation of hypothalamic neurons can inhibit the input of nociceptive signals to the pain centers in the cortex (Lin and Chen, 2008). CCI rat following EA intervention, there were 17 hypothalamic proteins identified with significant changes in the expression. EA attenuated pain may *via* regulation of expression of these proteins in the hypothalamus (Vogt, 2016). Although studies have indicated involvement of many brain structures in the modulation of acupuncture analgesia, hypothalamus might play a crucial role in this process. A study revealed that increased local synaptic activity in the ipsilateral somatosensory cortex and decreased in the contralateral somatosensory cortex after sciatic nerve injury. EA served as repeated sensory stimulation, might potentially induce increased synaptic activity in the corresponding cortices involving contralateral somatosensory cortex of sciatic nerve injury (Wu et al., 2018).

As can be seen, the descending pain modulation system, including the ACC, the PAG, the NAc, and the hypothalamus, plays an important role in EA analgesia. EA might reverse the maladaptive brain plasticity, promote the release of endogenous substances such as GABA, NE, 5-HT, and opioids. This could be an important mechanism underlying the post-therapeutic effect of EA, and it deserves further study.

## Discussion

Acupuncture, which has a history of 2,000-year, is a useful adjunct therapy or an acceptable alternative treatment of pain (Wells et al., 2017). The research base for acupuncture is rapidly expanding. Somatosensory stimulation, including acupuncture, could thus act as an additional input to re-arrange the neural loop, nociceptive, and acupuncture signals was integrated at spinal and supraspinal levels (Figure 4). Acupuncture suppressed pain, probably due to effects on the afferent nerve. The gate control theory might play a role in acupuncture analgesia. Non-painful input by acupuncture closed the “gates” to painful input, which prevented pain sensations from traveling to the central nervous system (Lemmon, 2018). Modulation of sensory input occurs at the primary afferent neuron and spinal dorsal horn during an acupuncture treatment, which may depend on acupoints at the same spinal section as the pain site. We speculated, in this case, acupuncture inhibited neuronal activity caused by neuropathic pain, through reducing pain-related ion channels and protein kinases activation (Figure 5). In the spinal dorsal horn, EA down-regulated the neuronal chemokine CX3CL1 and prevented microglial p38 MAPK/ERK signaling pathway, leading to reduced release of TNF-α and IL-1β, BDNF, PGE2, and reducing the neuronal excitability and inflammatory response (Figure 6). Furthermore, functional magnetic resonance imaging studies have shown that acupuncture at specific acupoints modulated areas of the brain (Huang et al., 2021), and acupuncture activated descending pain control system. The distal acupoints such as Neiting (ST 44), Hegu 4 (LI 4), and Sanjian (LI 3) probably work in this way. Research has found that, in nerve injury model, contralateral but not ipsilateral acupuncture produced clear analgesia. This difference might be due to the damaged nerve blocks the conduction of acupuncture signals, while contralateral acupuncture inhibited pain through the central descending inhibitory, independent of the influence of local damaged nerves (Zhang H. et al., 2018). GABA, NE, 5-HT, and endogenous opioids are involved centrally (Figure 7). Taken together, acupuncture, as a distinctive therapeutic modality to pain, produced physiologic changes in the brain, spinal cord, and at the periphery. Medication treatment was often associated with side effects, and many patients did not achieve adequate pain relief at tolerated doses (Finnerup et al., 2015). On the contrary, acupuncture is a relatively safe and well-tolerated treatment, with most patients experiencing no adverse effects at all (Pfister et al., 2010).

As a matter of fact, the optimal prescription of acupuncture treatment (acupuncture point, degree of stimulation, frequency of treatment, and a number of treatment sessions) for neuropathic pain is a controversial issue amongst acupuncture experts. EA and MA are two acupuncture manipulations commonly used in clinical practice. Our previous study found that EA and MA effectively improved pain symptoms in patients with osteoarthritis of the knee, but EA had a faster onset of action than MA (Xu et al., 2020). Current evidence does not yet support an efficacy difference between MA and EA in the treatment of neuropathic pain. MA involves inserting an acupuncture needle into an acupuncture point and then twisting it up and down with the hand. EA delivers an electric current to the acupoint through an inserted needle. In terms of “needling feeling,” EA is often described as painful and numb, while MA is dominated by heaviness and distension in the deep tissue beneath the acupuncture point. MA activated all types of afferent fibers (Aβ, Aδ, and C) (Kagitani et al., 2010). The current intensity of EA was sufficient to excite Aβ-and some Aδ-fibers inducing analgesic effects (Zhao, 2008). Reason for this difference might be that MA and EA activated distinct ion-channels, but this inference had not been confirmed. In clinical practice, the intense intensity of EA was not suitable for patient analgesia, because the excitation of C fibers by synchronous strong electrical pulses would inevitably cause unbearable pain. The parameters of the EA could be precisely characterized. A study characterized the generation and transmission of electrical signals in Aβ-and some Aδ-fibers induced by acupuncture-like stimuli. EA in frequency-specific modes (2/15 Hz or 2/100 Hz) best mimicked MA (Huo R. et al., 2020). The efficacy of MA and EA also may be influenced by disease state, acupuncture duration, acupuncture parameters and acupoints. Therefore, clarifying the analgesic mechanisms of MA and EA and selecting the appropriate acupuncture modality are essential to improve clinical efficacy.

The frequency-dependent study for analgesia at high-and low-frequency highlighted the best operating parameters. Low-frequency (2 Hz) EA caused the release of neuropeptides such as encephalin and endorphin, which acedt on mu and/or delta opioid receptors to mediate analgesia; high-frequency (100 Hz) EA caused the release of dynorphin, which was mediated by kappa opioid receptors to mediate analgesia. Certain brain regions have been found to be associated with the release of various types of central opioid peptides (Zhang et al., 2003), but it was unclear how these brain regions were modulated by the 2 Hz and 100 Hz EA, respectively. The studies screened frequencies of EA, and the results indicated that in rats with neuropathic pain, 2 Hz EA induced a robust and longer lasting analgesic than 100 Hz EA (He et al., 2017; Xia et al., 2019). In type 2 diabetic neuropathic pain rat, EA at both 2, and 100 Hz down-regulated CGRP (Calcitonin gene related peptide) and P2X3 receptors overexpression in DRGs, but the analgesic effect of EA was stronger at 2 Hz (He et al., 2017). Another study showed that compared with 100 Hz EA, 2 Hz EA effectively regulated the expression level of genes in the arcuate nucleus region of the hypothalamus, especially those related to neurogenesis (Wang et al., 2012).

Acupoints have a characteristic that they become sensitive and even painful when exposed to pathological processes (Zhou and Benharash, 2014). The analysis of anatomical have revealed that acupoints have a number of elements such as a high density of nerve endings, A-and C-afferent fibers and vascular, which could perceive stimulation (Li et al., 2004). When stimulating acupoints, the local of acupoints may release biomolecules to exert the role of analgesia or neuromodulation. Acupuncture stimulates the somatic afferent nerves of the skin and muscles under the acupoints. Then, the somatic sensory information is carried to the spinal cord and cortex area of the brain that modulate spinal signal transmission and pain perception in the brain (Wang et al., 2008). Therefore, acupuncture analgesia was essentially a manifestation of integrative processes at different levels of the nervous system between afferent impulses from the pain regions and impulses from acupoints. The infiltration of procaine, a local anesthetic, into the deep tissues around the point of acupuncture entirely abolished the analgesic effect, suggesting that nerves was mediators of this response (Zhou and Benharash, 2014). The selection of acupoints may make the effect of acupuncture more targeted in different diseases and different pain sites. But there are no studies to explain in the treatment of pain why acupoints are effective and non-acupoints are not, or why this acupoint is effective and other acupoints are not. Therefore, the specificity of the acupoints should be studied further.

Acupuncture has been used to treat neuropathic pain caused by different diseases. In addition to nerve injury-induced peripheral neuropathic pain, a meta-analysis showed that benefit for acupuncture over control in the treatment of neuropathic pain caused by diabetes, human immunodeficiency virus (HIV), Bell’s palsy, and carpal tunnel syndrome (Dimitrova et al., 2017). Here, it should be pointed out that diabetic neuropathy, a major complication of diabetes mellitus, refers to a collection of clinically diverse disorders affecting the nervous system. Despite most of diabetes peripheral neuropathy is characterized by hypoesthesia, it also may present with pain. Of all diabetic peripheral neuropathy patients, 20% develop neuropathic pain (Sloan et al., 2018). Acupuncture was considered as a treatment option for diabetic neuropathic pain. Zusanli (ST 36), Feishu (BL 13), Pishu (BL 20), Sanyinjiao (SP 6), and Yinlingquan (SP 9) were the most widely used acupoints (Cho and Kim, 2021). And acupuncture could have a beneficial effect on neurological and motor function recovery (Fan et al., 2018). Previous research indicated that acupuncture also appeared to improve motor and sensory nerve conduction parameters, curing the disease from both sensory and functional aspects (Dimitrova et al., 2017). In spinal cord injury patients, acupuncture could be useful to improve pain and other complications if patients experience side effects or have no (or a weak) response to a conventional treatment (Heo et al., 2013). However, the analgesic effect of acupuncture on neuropathic pain induced by spinal cord injury has an obvious selectivity, which depends on the location and type of pain, as well as the type of injury (Siddall and Middleton, 2015). Acupuncture recipients with incomplete damage to central nervous system pathways that remained intact appeared to recover better than those with complete damage, and patients with musculoskeletal pain responded better to treatment compared with those with central pain. In addition, participants with moderate pain were more likely to achieve long-term pain relief than those suffering from severe pain (Mehta et al., 2013).

In clinical and preclinical models, some forms of neuropathic pain persist as a result of sympathetic nerve activity, and local sympathetic blockade or lesion is used to treat it (Xie et al., 2016, 2020). After peripheral nerve injury, sympathetic neurons sprout within DRG, sensitizing nociceptive neurons to adrenergic stimulation, although this remains controversial (Hoffman et al., 2018). A new study provided evidence that sympathetic sprouting in the DRG played a role in spontaneous pain in the SNI and related neuropathic pain models (Zheng et al., 2022). They concluded that norepinephrine released from sympathetic induced DRG neuronal clustering discharge, which correlated directly with spontaneous pain behavior caused by nerve injury. Research has shown that acupuncture reduced sympathetic nerve hyperactivity (Li et al., 2009). However, EA for neuropathic pain by modulating sympathetic nerves has not been studied.

## Summary and future directions

In neuropathic pain conditions, acupuncture may improve pain through somatosensory system including both central and peripheral mechanism. In the periphery, acupuncture inhibited neuronal activity caused by neuropathic pain, through reducing pain-related ion channels and receptors activation (Figure 5). In the spinal dorsal horn, EA down-regulated the neuronal chemokine CX3CL1 and prevented microglial p38 MAPK/ERK signaling pathway, leading to reduced release of TNF-α and IL-1β, BDNF, PGE2, and reducing the neuronal excitability and inflammatory response (Figure 6). Furthermore, acupuncture activated descending pain control system (Figure 7). The cumulative evidence demonstrated that acupuncture provided an alternative or adjunctive therapy for neuropathic pain.

In traditional Chinese medicine, the choice of appropriate acupoints is the key to acupuncture treatment. Intensities, frequencies, and the course of treatment of acupuncture all affect analgesic effects. However, the differences between MA and EA, the effects of EA frequency on relevant brain regions and the possible systematic differences between acupoint and non-acupoint are currently unknown. Conducting these studies in the future will provide better evidence to guide clinical on acupuncture modalities, acupuncture parameters and acupoints for the treatment of neuropathic pain. In the light of addressing the above issues, future studies shall be conducted in a broader context. High-quality, multifaceted basic research that explores the mechanisms of acupuncture analgesia will provide more possibilities for pain management.

## Author contributions

XM, H-PL, and C-ZL put forward the idea of performing the review. XM wrote the initial manuscript. H-PL and C-ZL revised and edited the manuscript. WC and X-WH draw the manuscript. N-NY, LW, and C-XT summarized the tables. All authors have approved the submitted version.
